# Plant production of high affinity nanobodies that block SARS-CoV-2 spike protein binding with its receptor, human angiotensin converting enzyme

**DOI:** 10.3389/fbioe.2022.1045337

**Published:** 2022-12-23

**Authors:** Marco Pitino, Laura A. Fleites, Lauren Shrum, Michelle Heck, Robert G. Shatters

**Affiliations:** ^1^ AgroSource, Inc, Jupiter, FL, United States; ^2^ Emerging Pests and Pathogens Research Unit, USDA Agricultural Research Service, Ithaca, NY, United States; ^3^ U.S. Horticultural Research Laboratory, Subtropical Insects and Horticulture Research Unit, USDA Agricultural Research Service, Fort Pierce, FL, United States

**Keywords:** SARS-CoV-2, nanobody, plant, VHH, competitive ELISA, ACE2, biofactory, plant cell culture

## Abstract

Nanobodies^®^ (V_HH_ antibodies), are small peptides that represent the antigen binding domain, V_HH_ of unique single domain antibodies (heavy chain only antibodies, HcAb) derived from camelids. Here, we demonstrate production of V_HH_ nanobodies against the SARS-CoV-2 spike proteins in the solanaceous plant *Nicotiana benthamiana* through transient expression and their subsequent detection verified through western blot. We demonstrate that these nanobodies competitively inhibit binding between the SARS-CoV-2 spike protein receptor binding domain and its human receptor protein, angiotensin converting enzyme 2. There has been significant interest and a number of publications on the use of plants as biofactories and even some reports of producing nanobodies in plants. Our data demonstrate that functional nanobodies blocking a process necessary to initiate SARS-CoV-2 infection into mammalian cells can be produced in plants. This opens the alternative of using plants in a scheme to rapidly respond to therapeutic needs for emerging pathogens in human medicine and agriculture.

## 1 Introduction

As the human population grows and increases in global human interactions are realized, there is concern, and supportive evidence, that human pandemics will occur more frequently (Alberola-Ila et al., 2020; Soga et al., 2021; Riccaboni and Verginer, 2022). Therefore, we will need to respond rapidly with targeted therapeutics. Severe acute respiratory syndrome coronavirus 2 (SARS-CoV-2; *Nidovirales*, *Coronaviridae*) is the most recent example of a human pathogenic virus that induced such a global pandemic (Huang et al., 2020). COVID-19 disease results in a range of outcomes, ranging from asymptomatic infection to patient death. To date, global vaccinations for SARS-CoV-2 protections are underway, but additional treatments are needed to prevent infection among naïve and even vaccinated individuals. Tiered prevention efforts have been shown to reduce transmission and severity of disease outcome and recent advances in antibody therapies promise to function as a component of this strategy.

Coronaviruses are positive-sense, single-stranded RNA viruses with spherical virions bound by a membrane envelope. Inserted into the membrane envelope are ∼25 copies of the homotrimeric transmembrane spike glycoprotein (spike protein) with a receptor binding domain (RBD) that is responsible for entry into host cells (Wang et al., 2020; Jackson et al., 2022) *via* interaction with the protein angiotensin converting enzyme 2 (ACE2), the interaction which also determines the viral host range. Studies have shown a higher affinity for SARS-CoV-2 to ACE2 as compared to ACE1, further supporting its role in transmission and virulence (Samavati and Uhal 2020). Highly transmissible viral variants, such as Delta and Omicron variant, exhibit mutations in the RBD (Li et al., 2021) (Saxena et al., 2022), highlighting the importance of the RBD in host-virus interactions. Thus, interactions between ACE2 and the RBD are attractive targets for the development of novel anti-viral therapies.

Nanobodies represent a promising new therapy for the treatment of viral diseases, including COVID-19. A PubMed search for SARS-CoV-2 and nanobody brings up a total of 217 peer-reviewed publications. Nanobodies, also referred to as single variable domains on a heavy chain (V_HH_), are produced by animals in the camelid family, which include llamas and alpacas. Coined by the popular press as mini-antibodies (Deyev and Lebedenko 2009), these IgGs are less than 15 kDa (as compared to ∼150 kDa for conventional antibodies) and are comprised of an unpaired heavy-chain variable domain. Nanobodies have been reported to bind antigens with affinities equivalent to a conventional IgG (Gonzalez-Sapienza et al., 2017; Asaadi et al., 2021). Key features of nanobodies that make them attractive alternatives to conventional antibodies include their high affinity, specificity, solubility, thermostability and mobility (Bannas et al., 2017; Wang et al., 2021). Production of nanobodies is typically done by expression of the gene in bacteria, yeast, and mammalian hosts (Muyldermans 2013; De Munter et al., 2018); however, there is growing interest and scientific research on the use of plants as biofactories for biomolecule production with evidence indicating advantages in speed, cost, scalability and flexibility in comparison to other bioproduction systems (McDonald and Holtz 2020). In fact, functional nanobodies have been produced in plants (for review see Wang et al., 2021). Nanobodies are also under development for the control of at least two crop diseases: grapevine fanleaf virus in cultivated wine grapes (Yan et al., 2020), botrytis, and for detection against a range of other plant pathogens (Njeru and Kusolwa 2021).

We represent a team of agricultural scientists developing sustainable and biologically-based solutions to pathogens of economic importance in crop production. As part of this research, we developed a low-cost, plant-based method of producing proteins that could be used to solve agricultural pathogen problems in agricultural production settings. As a proof-of-concept, we describe the production of a RBD nanobody in a plant expression system. The benefits of producing therapeutics in plants justify considering plants to mass produce COVID-19 protein-based therapies.

## 2 Materials and methods

### 2.1 Construct design

A total of four constructs were designed for experimentation with plant expression of COVID-19 nanobodies. The methionine start codon of the high affinity SARS-CoV2 nanobody protein sequence (NIH-CoVnb-112; Esparza et al., 2020) was removed and replaced with a widely used N-terminal signal peptide sequence from an *Arabidopsis thaliana* chitinase (At3g12500) (Haseloff et al., 1997) (Berthold et al., 2019). Accumulation of apoplast-targeted monoclonal antibodies in tobacco has been shown to be greater as compared to cytosolic accumulated recombinant antibody (Schillberg et al., 1999); however, relative extraction efficiencies of intracellular *versus* extracellularly targeted nanobody constructs were not evaluated herein. A 6x histidine tag was incorporated at the C-terminus for purification and detection of the mature nanobody (SP-CoV19_his; Supplementary Figure S1). An analogous negative mutated control construct was also designed, such that the amino acids spanning the three complementarity determining regions (CDR1, CDR2, CDR3) of the native SARS-CoV2 nanobody sequence were scrambled using a random number generator to create SP-mCov19_his; (Supplementary Figure S2). Disruption of the CDR regions was expected to abolish the interaction with the receptor binding domain of the viral spike protein.

Two more variants of the SARS-CoV2 nanobody construct were made as fusions to monomeric enhanced green fluorescent protein coding sequence mEGFP (Zacharias et al., 2002): one with an N-terminal 6x histidine tag (SP-his_CoV19-GFP), and a second with a C-terminal 6x histidine tag (SP-CoV19_his-GFP; Supplementary Figure S2). This module was followed in both constructs by mEGFP, with the porcine teschovirus-1 2 A peptide (P2A, a ribosome skipping sequence) (Szymczak et al., 2004) inserted between the nanobody module and mEGFP sequence to allow CoV19 variants and mEGFP to be produced as separate proteins. All constructs were codon-optimized for expression in the Solanaceae using an online tool provided by Integrated DNA Technologies (IDT, Illinois, United States; https://www.idtdna.com/CodonOpt) prior to uploading to the online ordering portal for Codex DNA (La Jolla, CA). A 40bp span of nucleotide sequence homologous to the recipient vector pNANO (Supplementary Figures S1, S2) was added to the 5′ and 3’ ends of the constructs to enable cloning with the BioXP 3250 system (Codex DNA, La Jolla, CA). Construct nucleotide sequences are available in Supplementary File S1.

### 2.2 Construct generation and bacterial transformation

**FIGURE 1:**
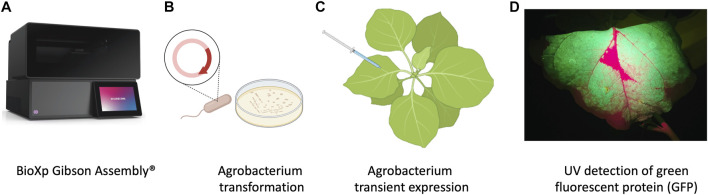
Schematic representation of workflow of production of nanobodies in plant system. **(A)** Cloning with the BioXP 3,250 Gibson Assembly^®^. **(B)** *Agrobacterium* transformation. **(C)** *Agrobacterium* infiltration was performed by using 1 mL needleless syringes to inject bacteria into the abaxial side of the leaves at OD_600_ 0.3. **(D)** P2A sequence was used for generating multiple separate proteins from a single mRNA, GFP included in the sequence allowed prescreening of high expression protein in leaves using UV light.

The plasmid backbone was linearized by sequential digestion with *Sma*I and *Spe*I (New England Biolabs, Ipswich, MA, United States), and gel purified from 0.8% SeaPlaque GTG Agarose (Lonza, Rockland, ME) using a phenol:chloroform:isoamyl alcohol extraction method followed by overnight precipitation at −20°C in 100% ethanol, 0.3 M NaOAc, pH 5.0. The purified, precipitated DNA was washed with 70% ethanol, dried briefly, and resuspended in sterile nuclease free water. The final constructs were generated in an overnight run on the BioXP 3250 system (Figure 1A) (Codex DNA, La Jolla, CA), an automated synthetic biology platform for DNA fragment assembly and cloning.

*Agrobacterium tumefaciens* EHA105 was electroporated with the BioXP products and grown on LB supplemented with kanamycin (100 mg/L) for 3 days (Figure 1B). Colonies were screened using colony PCR and sequence verified prior to transient expression and purification in *N. benthamiana*.

### 2.3 Plant growth and agroinfiltration

*N. benthamiana* plants were grown under the greenhouse conditions and used at 4–5 weeks old for transient expression using plant infiltration with *Agrobacterium* EHA105, which mechanically delivers the bacteria to the plant’s extracellular matrix (apoplast) (Kapila et al., 1997) (Figure 1C). *Agrobacterium* EHA105 harboring pNANO plasmid was cultured overnight in 5 mL of LB media with 100 mg/L of kanamycin. Overnight culture was pelleted and resuspended in infiltration buffer (10 mM MgCl2, 10 mM MES, 400 µM acetosyringone) at optical density at 600 nm (OD600) 0.3. For each construct, leaves were infiltrated with the bacterial suspension and set in greenhouse for duration of experiment (Figure 1C). Three plants for each construct and a total of 6 g of tissue was used 2 days post infiltration (2 dpi) when GFP fluorescent protein was readily visible under UV light, leaves were manually excised from the plants using a sterile blade and processed for total protein extraction and purification.

### 2.4 Protein extraction and purification

*N. benthamiana* leaves were observed under UV light for GFP expression and harvested at 2 dpi (days post infiltration) (Figure 1D) followed by homogenization in liquid nitrogen. Total plant proteins were extracted using native buffer (10 mM Tris/HCl pH 7.5, 150 mM NaCl, 0.5 mM EDTA, 1% [v/v] P9599 Protease Inhibitor Cocktail [Sigma-Aldrich], 1% [v/v] IGEPAL CA-630 [Sigma-Aldrich]). A total of 5 mL of extraction buffer per gram of leaf tissue was used. Samples were clarified by centrifugation at 4 C at 3,000 rcf. The supernatant was filtered through a 40 µm nylon cell strainer (Becton Dickinson Labware, Franklin Lakes, NJ, United States) and then used for purification process utilizing 3 ml Ni-NTA agarose columns (Thermo Scientific, Rockford, United States), following manufacturers guidelines. Briefly, imidazole binding buffer (20 mM sodium phosphate, 10 mM imidazole, 0.5 mM NaCl, pH 7.4) was used to equilibrate, bind, and wash the columns. To elute product of interest, (20 mM sodium phosphate, 500 mM imidazole, 0.5 mM NaCl, pH 7.4) was used.

### 2.5 SDS-PAGE and western blotting

Samples were denatured and reduced using 5x Lane Marker Reducing Sample Buffer (Thermo Scientific, Rockford, IL, United States), boiled at 95°C for 10 min, then stored on ice. Gradient 4%–20% precast polyacrylamide gels (Bio-Rad Laboratories, Hercules, CA) were loaded into electrophoresis tank (Bio-Rad Laboratories) and filled with 1x Tris/*Glycine*/SDS buffer. Kaleidoscope ladder (Bio-Rad Laboratories) was loaded into the first well (5 µL), and each sample was loaded into every other well. 25 µL per sample were used for Coomassie staining, and 10 µL per sample were used for immunoblotting. Electrophoresis was run following manufacturing guidelines with a powerpack (Bio-Rad Laboratories). One gel was stained with Coomassie blue, while the other gel was transferred to a nitrocellulose membrane using the Trans-Blot Turbo Transfer system following manufacturer guidelines (Bio-Rad Laboratories). The nitrocellulose membrane was removed and placed in 1X Casein blocker for 1 hour on rotator followed by incubation with a 1:1,000 dilution of his HRP-conjugated antibodies (Proteintech, Rosemount, IL, United State) for 1 h room temperature. The membrane was washed in 1X TBS three times in 10 min intervals. ECL substrate (Bio-rad Laboratories) that consists of 1 mL peroxide and 1 mL luminol enhancer were spread onto membrane and left for 5 min before observation using ChemiDoc imager (Bio-rad Laboratories).

### 2.6 Competitive ELISA binding screen for ACE2 and RBD

To verify the activity of recombinant nanobodies generated in plants, we conducted a competitive binding assay that measures inhibition of the interaction between the receptor binding domain (RBD) of the SARS-CoV-2 spike protein with the ACE2 receptor in the presence of the purified nanobodies. Purified nanobodies were diluted at 1 μg/mL and 0.1 μg/mL concentrations, assessed by a Bradford assay, in association with RBD proteins then added to the ACE2 coated plate (RayBiotech, Peachtree Corners, GA, United State). Nanobodies and RBD proteins were incubated at room temperature for 1 hour to allow interaction. The assay plate was washed four times with a wash solution provided by ELISA kit (RayBiotech). HRP-conjugated Anti-IgG was added to plate post wash and incubated at room temperature for 1 hour with gentle shaking. After four additional washes, the plate was developed by addition of tetramethylbenzidine and stopped after 30 min of gentle shaking in the dark with stop solution (RayBiotech). Absorbency was measured immediately after adding stop solution at 450 nm on a plate reader (Citation 1 imaging reader, BioTek, Winooski, VT, United States).

## 3 Results

### 3.1 Expression and purification of nanobodies for SARS-cov-2 RBD in *N. benthamiana*

**FIGURE 2:**
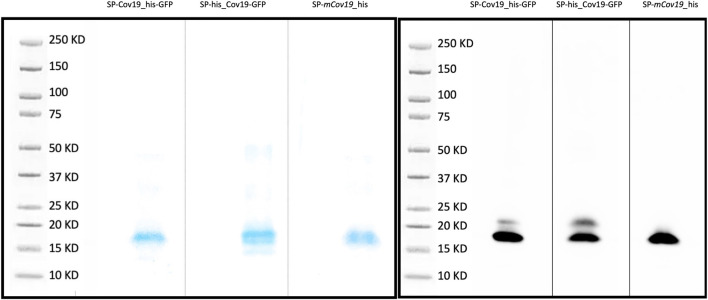
SDS PAGE and Western blot. **(A)** Coomassie blue stain was used to verify purity of concentrated proteins and band size. **(B)** Western blotting was carried out to detect the target purified SP-CoV19_his-GFP, SP-his_CoV19-GFP and negative control SP-mCov19_his using his antibodies.

An initial test performed using SP-CoV19_his purified from transient expressing leaves showed an expected ∼15 kDa band confirming expression and purification. This band was visualized by Coomassie blue staining of an SDS-Page gel and western blot/immunodetection specific for the his-tag on the SP-CoV19 protein (Supplementary Figure S3). Next, we tested SP-his_CoV19-GFP, SP-CoV19_his-GFP, SP-mCov19_his sequences. GFP visualization of infiltrated leaves showed high levels of expression 2 days post infiltration and leaves were harvested at this time and used for purification. Bands on the Coomassie gel and western blot were visualized migrating between the 15 and 20 kDa marker bands corresponding to the size of the SP-his_CoV19-GFP (17.21 kDa mature protein 19.45 kDa unprocessed protein), SP-CoV19_his-GFP (17.21 kDa mature protein 19.45 kDa unprocessed protein) and SP-mCov19 his sequences (15.14 kDa mature protein 17.38 kDa unprocessed protein) (Figures 2A, B).

### 3.2 Biological activity of SARS-Cov-2 nanobody with ACE2 competition assay

**FIGURE 3:**
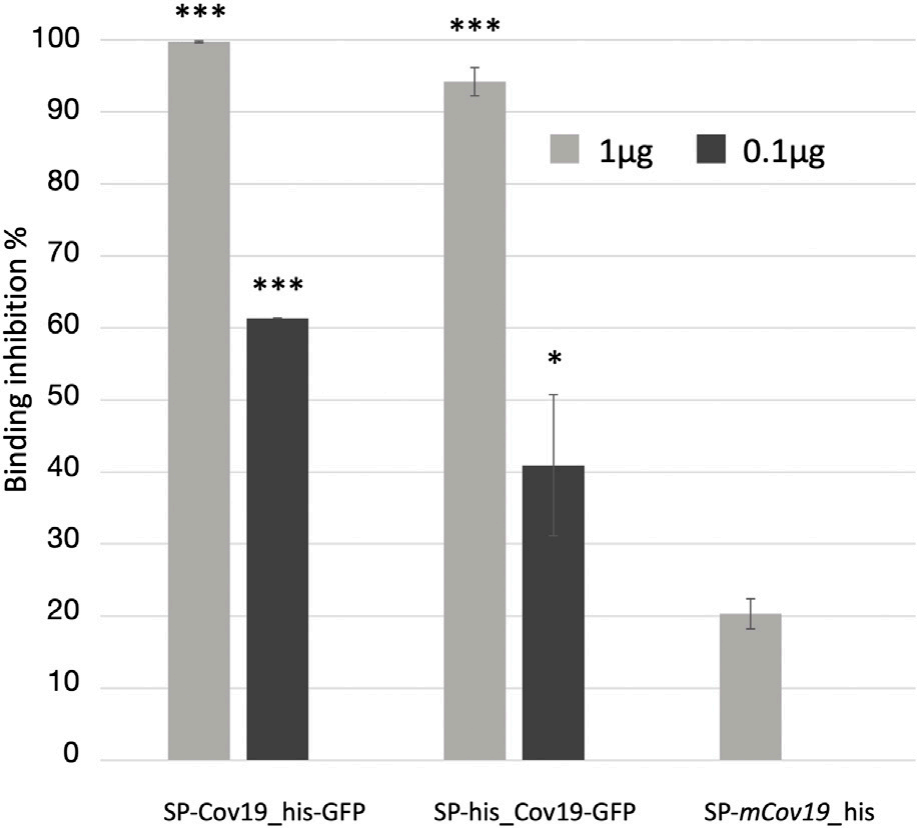
Competitive ELISA inhibition of ACE2 and RBD binding using anti RBD nanobodies. Competition binding assays were used to investigate whether the SP-Cov19_his-GFP and SP-his_CoV19-GFP blocked the binding of RBD to ACE2 compared to the mutant version SP-*mCov19*_his. SP-CoV19_his-GFP, SP-his_CoV19-GFP and SP-mCov19_his at 1 μg/mL and 0.1 μg/mL were incubated with RBD proteins, both SP-CoV19_his-GFP, SP-his_CoV19-GFP inhibited RBD bound to ACE-2 but SP-mCoV19_his at 1 μg/mL (Bars represent standard deviation, the asterisks indicate significant differences between conditions, * = *p* < .05, ** = *p* < .005, *** = *p* < .001).

Next, we assessed the ability of the plant produced nanobody to block ACE2 and RBD protein interaction. To evaluate relative inhibition of RBD protein from binding to ACE2, a competitive ELISA inhibition assay was performed. RBD protein binding ACE2 was indicated by high colorimetric absorbance. Initial screening was performed using SP-CoV19_his at 100, 10, 1 and 0.1 μg/mL concentrations (Supplementary Figure S3C). Competitive ELISA assay indicated that 1 μg/mL SP-CoV19_his inhibited interaction between ACE2 and RBD, and this concentration was used for subsequent experiments. The same results were obtained using both SP-his_CoV19-GFP and SP-CoV19_his-GFP, showing 100% inhibition between ACE2 and RBD at 1 μg/ml. Inhibition was also observed at 0.1 μg/ml with 60%–70% inhibition (Figure 3). In contrast, the mutated sequence SP-*mCov19*_his inhibited less than <20% at 1.0 μg/ml and 0% at 0.1 μg/ml (Figure 3). These results showed that plant-produced SP-CoV19_his, SP-his_CoV19-GFP and SP-CoV19_his-GFP, but not SP-*mCov19*_his, inhibit 100% ACE2 and RBD interactions at 1 μg/mL similarly to previous published data with NIH-CoVnb-112 production in a yeast system (Esparza et al., 2020). A two-sample *t*-test was applied to compare the groups, the asterisks indicate significant differences among conditions, * = *p* < .05, ** = *p* < .005, *** = *p* < .001) (Figure 3).

## 4 Discussion

Research using nanobodies in plants has been increasing rapidly in recent years (Dhama et al., 2020; Wang et al., 2021), including in the development of therapies and diagnostic tools for plant diseases. Plants offer several advantages for nanobodies expression over conventional expression platforms including their easy transformation, low risk of pathogen contamination and low cost for upscaling (Orzaez et al., 2009; Tschofen et al., 2016). In addition to injectable vaccines, new strategies are emerging and being developed to increase protection against COVID-19, for example a nasal spray-delivered nanobody offers a complementary barrier method to prevent virus acquisition into human epithelial cells in the airway. Nanobodies are 12–15 kDa, single-domain antibody fragments that can be delivered by nebulizers and relatively easy and inexpensive to produce compared to other systems (Esparza et al., 2020). Previous cryo-electron microscopy studies showed SARS-CoV-2 spike protein and its interaction with the cell receptor ACE2, such binding triggers a cascade of events that leads to the fusion between cell and viral membranes for cell entry (Kirchdoerfer et al., 2018; Song et al., 2018; Ni et al., 2021; Zhu et al., 2021). Because SARS-CoV-2 binding spike protein RBD and the host ACE2 receptor determines host susceptibility to the virus, interfering with that interaction might constitute a treatment option (Walls et al., 2020).

In this study, we provide proof of concept for *in-planta* production of nanobodies that neutralize the interaction between the human ACE2 receptor, and the SARS-CoV-2 spike protein RBD, a key step of the infection initiation process (Du et al., 2009; Esparza et al., 2020; Huang et al., 2020; Jawad et al., 2021; Ou et al., 2021). A SARS-CoV-2/ACE2 binding inhibition assay was performed and demonstrated that the plant-produced SARS-CoV-2 spike protein nanobody inhibited the SARS-CoV-2 binding to the ACE receptor similarly to that reported earlier for the same yeast-produced nanobody (Esparza et al., 2020). Moreover, a modified nanobody with a scrambled RBD binding domain did not demonstrate the inhibition ability observed with the spike protein-targeted antibody, demonstrating the binding specificity of the interaction between the plant-produced RBD-binding nanobodies and the RBD. A recent example of production of RBD *in planta* exhibits suitable biochemical and antigenic features for use in a subunit vaccine platform (Dhama et al., 2020; Demone et al., 2021; Maharjan et al., 2021; Mamedov et al., 2021; Mardanova et al., 2021; et al., 2021; Ceballo et al., 2022). Thus, scientists have shown that both the RBD antigen and RBD-specific antibodies can be generated in plants [Also see: (Diego-Martin et al., 2020)]. Several molecular features engineered into our plasmid will facilitate future experiments and scale up of the plant-produced nanobody. The plant expression constructs we used include two features to aid in nanobody production: the use of a well-characterized signal peptide targeting the nanobody to the plant apoplastic space and a self-cleaving P2A peptide. A signal peptide was added for future nanobody production in plant cell tissue culture systems, to support secretion of the nanobody through the cellular secretory pathway. The self-cleaving, P2A peptide induces ribosome skipping during protein translation which results in the production of two discrete proteins from a single mRNA molecule (Szymczak et al., 2004). The P2A sequence inserted between the nanobody and GFP enabled production of functional nanobodies with the separate and concurrent production of GFP used as a fluorescent signal to monitor transient transformation events in *N. benthamiana* and to easily select nanobody producing cells based on fluorescence when cells are placed into culture to select stable transformation events. The His tag facilitated the nanobody enrichment from the background of the plant proteome.

Herein, we demonstrate that nanobodies produced in plants retain proper folding and functionality as previously reported in a yeast production system (Esparza et al., 2020), thus supporting the use of plants as cost-effective production platforms for therapeutic needs for emerging pathogens, such as the SARS-CoV-2 virus. Biomolecule production is expected to exceed USD 100 billion in the next few years (Zarrelli 2022), and we posit that plant molecular farming of nanobodies, and other biologicals is an under-developed area that could support this market. Plant-based biomanufacturing technologies are currently being developed and improved, and use of these systems for biomolecule production is expected to grow substantially in the near future, especially with field grown plants that only require light, water and fertilizer (McDonald and Holtz 2020). Scalability of biomolecule production in plant cells from plant cell culture, to greenhouse and even field grown plants make plant-based biofactories an attractive alternative. This concept of plant molecular farming has been described in numerous papers (for recent reviews see (Buyel et al., 2021; De Martinis et al., 2022). While a few perceived limitations of molecular farming exist (Schillberg and Finnern 2021), including low productivity of plants, costs of downstream processing, and slow translation to applications, new and emerging plant biotechnology, such as the rapid transient expression methods described in this paper and plant Symbiont™ technology (Shatters et al., 2021), will certainly advance the field and create new market possibilities for biomolecule production in plants to meet the challenges raised by of emerging pests an pathogens moving forward.

## Data Availability

The original contributions presented in the study are included in the article/Supplementary Material, further inquiries can be directed to the corresponding author.

## References

[B44] Alberola-Ila E. Arslan Y. Cheng G. Moessner R. (2020). “The fiscal response to the Covid-19 crisis in advanced and emerging market economies (No. 23),“ in Bank for International Settlements.

[B1] Asaadi Y. Jouneghani F. F. Janani S. Rahbarizadeh F. (2021). A comprehensive comparison between camelid nanobodies and single chain variable fragments. Biomark. Res. 9 (1), 87. 10.1186/s40364-021-00332-6 34863296 PMC8642758

[B2] Bannas P. Hambach J. Koch-Nolte F. (2017). Nanobodies and nanobody-based human heavy chain antibodies as antitumor therapeutics. Front. Immunol. 8, 1603. 10.3389/fimmu.2017.01603 29213270 PMC5702627

[B3] Berthold F. Roujol D. Hemmer C. Jamet E. Ritzenthaler C. Hoffmann L. (2019). Inside or outside? A new collection of gateway vectors allowing plant protein subcellular localization or over-expression. Plasmid 105, 102436. 10.1016/j.plasmid.2019.102436 31449836

[B4] Buyel J. F. Stöger E. Bortesi L. (2021). Targeted genome editing of plants and plant cells for biomanufacturing. Transgenic Res. 30 (4), 401–426. 10.1007/s11248-021-00236-z 33646510 PMC8316201

[B5] Ceballo Y. López A. González C. E. Ramos O. Andújar I. Martínez R. U. (2022). Transient production of receptor-binding domain of SARS-CoV-2 in Nicotiana benthamiana plants induces specific antibodies in immunized mice. Mol. Biol. Rep. 49, 6113–6123. 10.1007/s11033-022-07402-4 35526244 PMC9079970

[B6] De Martinis D. Hitzeroth II MatsudaSoto R. N. Pérez Benvenuto E. (2022). Editorial: Engineering the plant biofactory for the production of biologics and small-molecule medicines-volume 2. Front. Plant Sci. 13, 942746. 10.3389/fpls.2022.942746 35873996 PMC9301360

[B7] De Munter S. Ingels J. Goetgeluk G. Bonte S. Pille M. Weening K. (2018). Nanobody based dual specific CARs. Int. J. Mol. Sci. 19 (2), 403. 10.3390/ijms19020403 29385713 PMC5855625

[B8] Demone J. Nourimand M. Maltseva M. Nasr-Sharif M. Galipeau Y. Langlois M.-A. (2021). Plant-based production of SARS-CoV-2 antigens for use in a subunit vaccineJordan Demone, Maryam Nourimand, Mariam Maltseva, Mina Nasr-Sharif, Yannick Galipeau, Marc-André Langlois, Allyson M. MacLean. bioRxiv. 10.1101/2021.10.17.464700 PMC9749978 36516116

[B9] Deyev S. M. Lebedenko E. N. (2009). Modern technologies for creating synthetic antibodies for clinical application. Acta Naturae 1 (1), 32–50. 10.32607/20758251-2009-1-1-32-50 22649585 PMC3347500

[B10] Dhama K. Natesan S. Iqbal Yatoo M. Patel S. K. Tiwari R. Saxena S. K. (2020). Plant-based vaccines and antibodies to combat COVID-19: Current status and prospects. Hum. Vaccin. Immunother. 16 (12), 2913–2920. 10.1080/21645515.2020.1842034 33270484 PMC7754927

[B11] Diego-Martin B. González B. Vazquez-Vilar M. Selma S. Mateos-Fernández R. Gianoglio S. (2020). Pilot production of SARS-CoV-2 related proteins in plants: A proof of concept for rapid repurposing of indoor farms into biomanufacturing facilities. Front. Plant Sci. 11, 612781. 10.3389/fpls.2020.612781 33424908 PMC7785703

[B12] Du L. He Y. Zhou Y. Liu S. Zheng B.-J. Jiang S. (2009). The spike protein of SARS-CoV — A target for vaccine and therapeutic development. Nat. Rev. Microbiol. 7 (3), 226–236. 10.1038/nrmicro2090 19198616 PMC2750777

[B13] Esparza T. J. Martin N. P. Anderson G. P. Goldman E. R. Brody D. L. (2020). High affinity nanobodies block SARS-CoV-2 spike receptor binding domain interaction with human angiotensin converting enzyme. Sci. Rep. 10 (1), 22370. 10.1038/s41598-020-79036-0 33353972 PMC7755911

[B14] Gonzalez-Sapienza G. Rossotti M. A. Tabares-da Rosa S. (2017). Single-domain antibodies as versatile affinity reagents for analytical and diagnostic applications. Front. Immunol. 8, 977. 10.3389/fimmu.2017.00977 28871254 PMC5566570

[B15] Haseloff J. Siemering K. R. Prasher D. C. Hodge S. (1997). Removal of a cryptic intron and subcellular localization of green fluorescent protein are required to mark transgenic Arabidopsis plants brightly. Proc. Natl. Acad. Sci. U. S. A. 94 (6), 2122–2127. 10.1073/pnas.94.6.2122 9122158 PMC20051

[B16] Huang C. Wang Y. Li X. Ren L. Zhao J. Hu Y. (2020). Clinical features of patients infected with 2019 novel coronavirus in Wuhan, China. Lancet 395 (10223), 497–506. 10.1016/s0140-6736(20)30183-5 31986264 PMC7159299

[B17] Huang Y. Yang C. Xu X. F. Xu W. Liu S. W. (2020). Structural and functional properties of SARS-CoV-2 spike protein: Potential antivirus drug development for COVID-19. Acta Pharmacol. Sin. 41 (9), 1141–1149. 10.1038/s41401-020-0485-4 32747721 PMC7396720

[B18] Jackson C. B. Farzan M. Chen B. Choe H. (2022). Mechanisms of SARS-CoV-2 entry into cells. Nat. Rev. Mol. Cell Biol. 23 (1), 3–20. 10.1038/s41580-021-00418-x 34611326 PMC8491763

[B19] Jawad B. Adhikari P. Podgornik R. Ching W.-Y. (2021). Key interacting residues between RBD of SARS-CoV-2 and ACE2 receptor: Combination of molecular dynamics simulation and density functional calculation. J. Chem. Inf. Model. 61 (9), 4425–4441. 10.1021/acs.jcim.1c00560 34428371

[B20] Kapila J. De Rycke R. Van Montagu M. Angenon G. (1997). An Agrobacterium-mediated transient gene expression system for intact leaves. Plant Sci. 122 (1), 101–108. 10.1016/s0168-9452(96)04541-4

[B21] Li J. Lai S. Gao G. F. Shi W. (2021). The emergence, genomic diversity and global spread of SARS-CoV-2. Nature 600 (7889), 408–418. 10.1038/s41586-021-04188-6 34880490

[B47] Kirchdoerfer R. N. Wang N. Pallesen J. Wrapp D. Turner H. Cottrell C. (2018). Stabilized coronavirus spikes are resistant to conformational changes induced by receptor recognition or proteolysis. Sci. Rep. 8, 15701. 10.1038/s41598-018-34171-7 30356097 PMC6200764

[B22] Maharjan P. M. Cheon J. Jung J. Kim H. Lee J. Song M. (2021). Plant-expressed receptor binding domain of the SARS-CoV-2 spike protein elicits Humoral immunity in mice. Vaccines 9 (9), 978. 10.3390/vaccines9090978 34579215 PMC8472882

[B23] Mamedov T. Yuksel D. Ilgın M. Gürbüzaslan I. Gulec B. Mammadova G. (2021). Production and characterization of nucleocapsid and RBD Cocktail antigens of SARS-CoV-2 in *Nicotiana benthamiana* plant as a vaccine candidate against COVID-19. Vaccines 9 (11), 1337. 10.3390/vaccines9111337 34835268 PMC8621474

[B24] Mardanova E. S. Kotlyarov R. Y. Ravin N. V. (2021). High-yield production of receptor binding domain of SARS-CoV-2 linked to bacterial flagellin in plants using self-replicating viral vector pEff. Plants 10 (12), 2682. 10.3390/plants10122682 34961153 PMC8708900

[B25] McDonald K. A. Holtz R. B. (2020). From farm to finger prick—a perspective on how plants can help in the fight against COVID-19. Front. Bioeng. Biotechnol. 8, 782. 10.3389/fbioe.2020.00782 32714921 PMC7351482

[B26] Muyldermans S. (2013). Nanobodies: Natural single-domain antibodies. Annu. Rev. Biochem. 82, 775–797. 10.1146/annurev-biochem-063011-092449 23495938

[B48] Ni W. Yang X. Yang D. Bao J. Li R. Xiao Y. (2013). Role of angiotensin-converting enzyme 2 (ACE2) in COVID-19. Crit Care 24 (1), 422. 10.1186/s13054-020-03120-0 PMC7356137 32660650

[B27] Njeru F. N. Kusolwa P. M. (2021). Nanobodies: Their potential for applications in biotechnology, diagnosis and antiviral properties in africa; focus on application in agriculture. Biotechnol. Biotechnol. Equip. 35 (1), 1331–1342. 10.1080/13102818.2021.1974943

[B28] Orzaez D. Granell A. Blazquez M. A. (2009). Manufacturing antibodies in the plant cell. Biotechnol. J. 4 (12), 1712–1724. 10.1002/biot.200900223 20014227

[B29] Ou J. Zhou Z. Dai R. Zhang J. Zhao S. Wu X. (2021). V367F mutation in SARS-CoV-2 spike RBD emerging during the early transmission phase enhances viral infectivity through increased human ACE2 receptor binding affinity. J. Virol. 95 (16), e0061721. 10.1128/jvi.00617-21 34105996 PMC8373230

[B46] Riccaboni M. Verginer L. Dai R. (2022). The impact of the COVID-19 pandemic on scientific research in the life sciences. PLOS ONE 17 (2), e0263001. 10.1371/journal.pone.0263001 35139089 PMC8827464

[B30] Samavati L. Uhal B. D. (2020). ACE2, much more than just a receptor for SARS-COV-2. Frontiers in Cellular and Infection Microbiology 10, 317. 10.3389/fcimb.2020.00317 32582574 PMC7294848

[B31] Saxena S. K. Kumar S. Ansari S. Paweska J. T. Maurya V. K. Tripathi A. K. (2022). Characterization of the novel SARS-CoV-2 Omicron (B.1.1.529) variant of concern and its global perspective. J. Med. Virol. 94 (4), 1738–1744. 10.1002/jmv.27524 34905235

[B32] Schillberg S. Finnern R. (2021). Plant molecular farming for the production of valuable proteins–Critical evaluation of achievements and future challenges. J. Plant Physiology 258, 153359. 10.1016/j.jplph.2020.153359 33460995

[B33] Schillberg S. Zimmermann S. Voss A. Fischer R. (1999). Apoplastic and cytosolic expression of full-size antibodies and antibody fragments in *Nicotiana tabacum* . Transgenic Res. 8 (4), 255–263. 10.1023/a:1008937011213 10621973

[B34] Shatters R. G. Stover E. Niedz R. L. Heck M. L. Pitino M. Grando M. F. (2021). Composition and methods for modifying a plant characteristic without modifying the plant genome. U. S. P. T. Office. USA: USDA Agricultural Research Service and AgroSource, Inc.

[B35] Siriwattananon K. Manopwisedjaroen S. Shanmugaraj B. Rattanapisit K. Phumiamorn S. Sapsutthipas S. (2021). Plant-produced receptor-binding domain of SARS-CoV-2 elicits potent neutralizing responses in mice and non-human primates. Front. Plant Sci. 12, 682953. 10.3389/fpls.2021.682953 34054909 PMC8158422

[B45] Soga M. Evans M. J. Cox D. T. C. Gaston K. J. (2021). Impacts of the COVID-19 pandemic on human–nature interactions: Pathways, evidence and implications. People Nat. 3, 518–527. 10.1002/pan3.10201 PMC8251160 34230912

[B49] Song W. Gui M. Wang X. Xiang Y. (2018). Cryo-EM structure of the SARS coronavirus spike glycoprotein in complex with its host cell receptor ACE2. PLoS Pathog. 14 (8), e1007236. 10.1371/journal.ppat.1007236 30102747 PMC6107290

[B36] Szymczak A. L. Workman C. J. Wang Y. Vignali K. M. Dilioglou S. Vanin E. F. (2004). Correction of multi-gene deficiency *in vivo* using a single 'self-cleaving' 2A peptide–based retroviral vector. Nat. Biotechnol. 22 (5), 589–594. 10.1038/nbt957 15064769

[B37] Tschofen M. Knopp D. Hood E. Stöger E. (2016). Plant molecular farming: Much more than medicines. Annu. Rev. Anal. Chem. Palo. Alto. Calif. 9 (1), 271–294. 10.1146/annurev-anchem-071015-041706 27049632

[B38] Walls A. C. Park Y. J. Tortorici M. A. Wall A. McGuire A. T. Veesler D. (2020). Structure, function, and antigenicity of the SARS-CoV-2 spike glycoprotein. Cell 181 (2), 281–292.e6. 10.1016/j.cell.2020.02.058 32155444 PMC7102599

[B39] Wang Q. Zhang Y. Wu L. Niu S. Song C. Zhang Z. (2020). Structural and functional basis of SARS-CoV-2 entry by using human ACE2. Cell 181 (4), 894–904. e899.e9. 10.1016/j.cell.2020.03.045 32275855 PMC7144619

[B40] Wang W. Yuan J. Jiang C. (2021). Applications of nanobodies in plant science and biotechnology. Plant Mol. Biol. 105 (1-2), 43–53. 10.1007/s11103-020-01082-z 33037986 PMC7547553

[B41] Yan R. Zhang Y. Li Y. Xia L. Guo Y. Zhou Q. (2020). Structural basis for the recognition of SARS-CoV-2 by full-length human ACE2. Science 367 (6485), 1444–1448. 10.1126/science.abb2762 32132184 PMC7164635

[B42] Zacharias D. A. Violin J. D. Newton A. C. Tsien R. Y. (2002). Partitioning of lipid-modified monomeric GFPs into membrane microdomains of live cells. Science 296 (5569), 913–916. 10.1126/science.1068539 11988576

[B43] Zarrelli A. (2022). Plants as biofactories to produce Food, medicines, and materials for a true green revolution. Int. J. Mol. Sci. 23, 5827. 10.3390/ijms23105827 35628636 PMC9145824

[B50] Zhu X. Mannar D. Srivastava S. S. Berezuk A. M. Demers J. P. Saville J. W. (2021). Cryo-electron microscopy structures of the N501Y SARS-CoV-2 spike protein in complex with ACE2 and 2 potent neutralizing antibodies. PLoS Biol. 19 (4), e3001237. 10.1371/journal.pbio.3001237 33914735 PMC8112707

