# Supplementary figures and images for: Plant production of high affinity nanobodies that block SARS-CoV-2 spike protein binding with its receptor, human angiotensin converting enzyme
Source: Front Bioeng Biotechnol. 2022 Dec 23;10:1045337. doi: 10.3389/fbioe.2022.1045337 (PMC9822723; doi:10.3389/fbioe.2022.1045337)

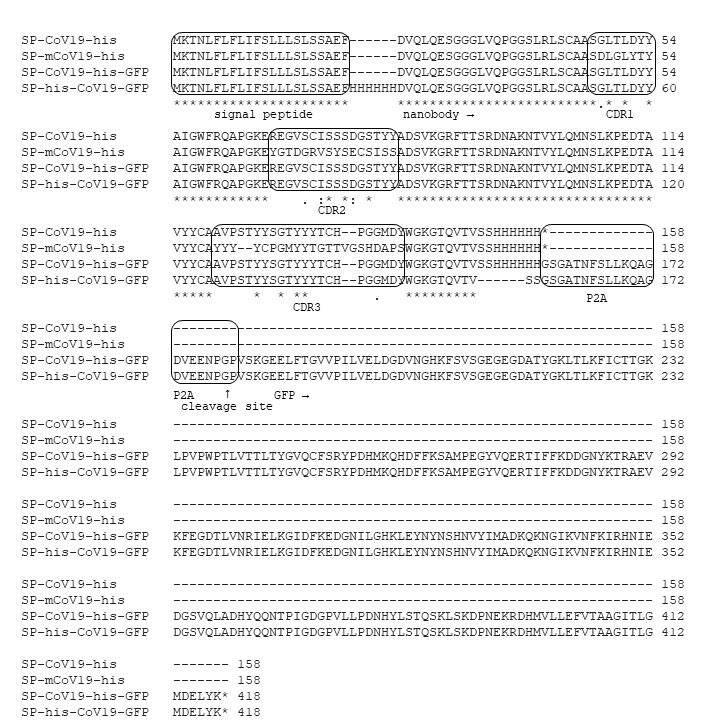

Supplement: Supplementary file 1 [file Image3.TIFF]

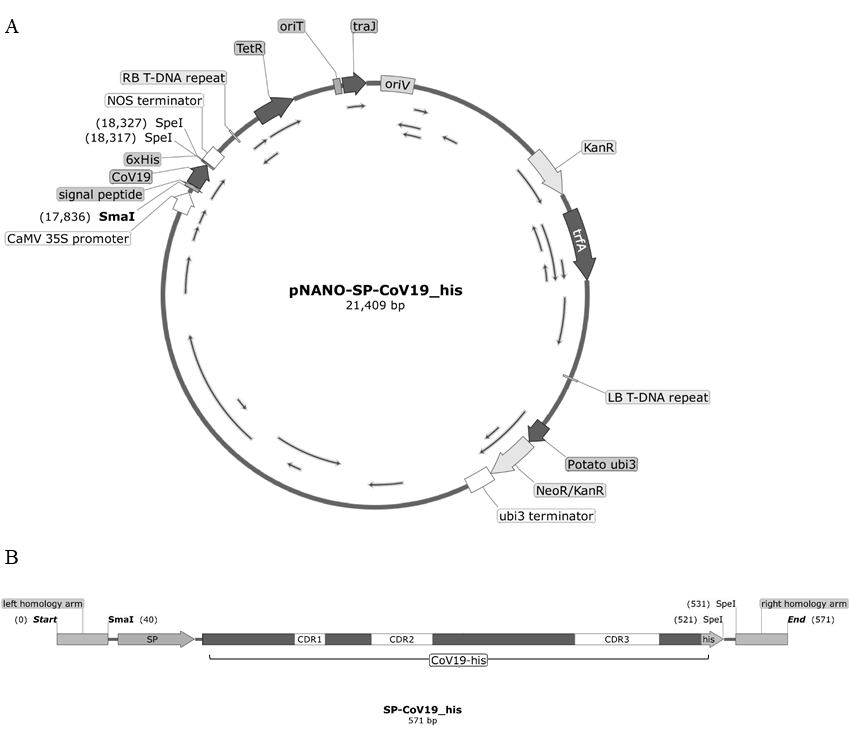

Supplement: Supplementary file 2 [file Image1.TIF]

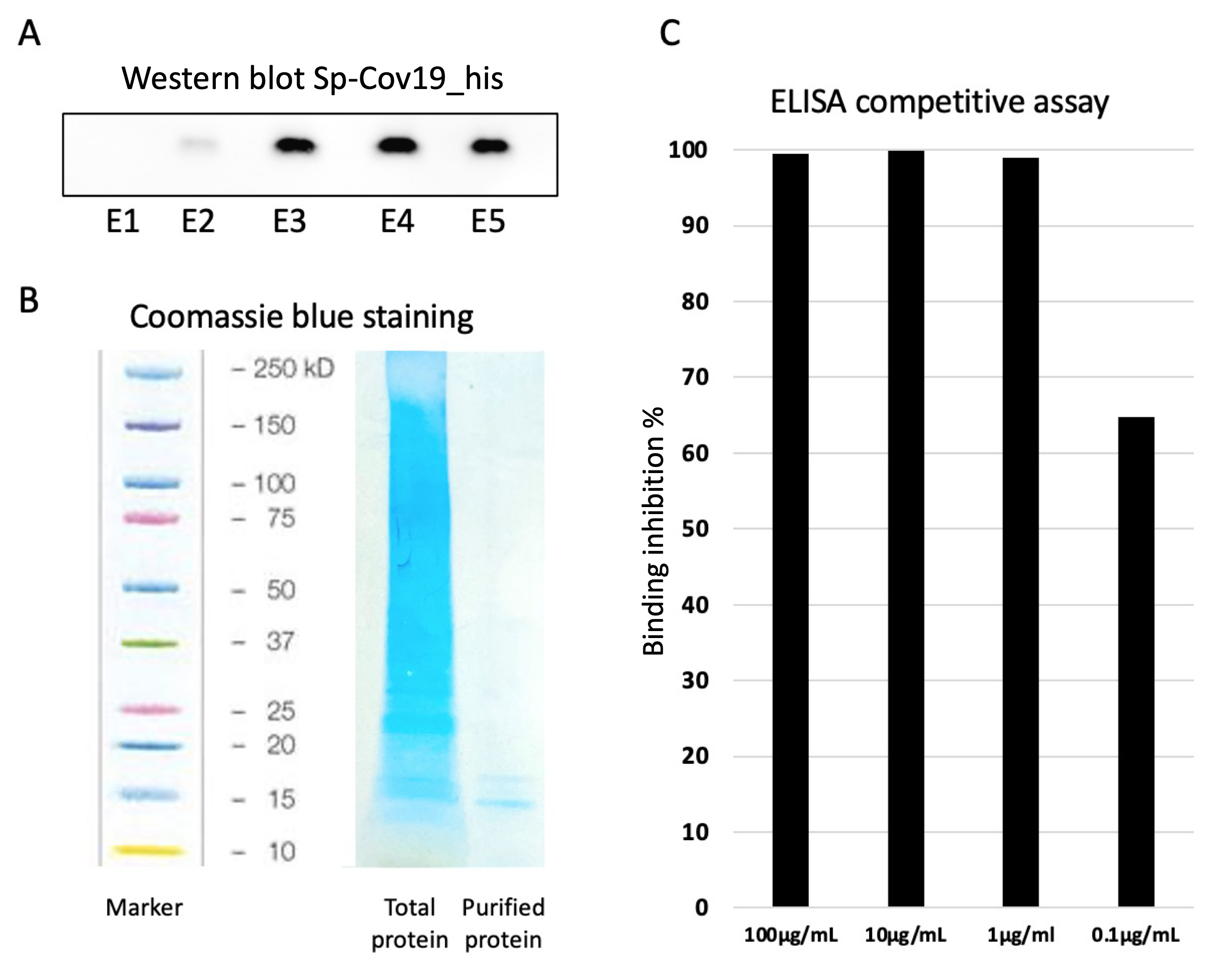

Supplement: Supplementary file 3 [file Image2.TIFF]
