# Supplementary material for: Plant production of high affinity nanobodies that block SARS-CoV-2 spike protein binding with its receptor, human angiotensin converting enzyme
Source: Front Bioeng Biotechnol. 2022 Dec 23;10:1045337. doi: 10.3389/fbioe.2022.1045337 (PMC9822723; doi:10.3389/fbioe.2022.1045337)
Supplement: Supplementary file 4 [file DataSheet1.DOCX]

Supplementary file 1. Nucleotide sequences of the various SARS-CoV19 nanobody constructs. Coding sequences are in capital letters. Homologous ends for cloning into pNANO on the BioXP 3250 are underlined.

>SP-CoV19_his

tggtttttatttttaattttctttcaaatacttccatcccgggaaaaaATGAAGACTAATCTTTTTCTCTTTCTCATCTTTTCACTTCTCCTATCATTATCCTCGGCCGAATTCGATGTCCAGTTACAGGAGTCAGGCGGGGGTCTGGTCCAGCCCGGAGGCAGCCTGCGTTTGTCCTGTGCAGCTTCAGGGCTAACACTTGATTACTATGCCATAGGGTGGTTCAGGCAAGCTCCAGGGAAAGAAAGGGAGGGGGTTAGTTGCATCTCATCCTCAGATGGCTCAACCTATTATGCTGACTCCGTTAAGGGCCGATTCACTACAAGCCGTGACAATGCCAAAAATACAGTCTACCTGCAGATGAATAGCCTCAAACCCGAAGACACTGCTGTGTACTATTGCGCCGCCGTACCATCCACCTATTACAGCGGCACATACTATTATACCTGCCACCCAGGTGGTATGGATTATTGGGGGAAAGGTACACAAGTAACTGTCAGCAGCCATCACCATCATCACCACTAGTGATAActagtgatcgttcaaacatttggcaataaagtttcttaag

>SP-*mCoV19*_his

tggtttttatttttaattttctttcaaatacttccatcccgggaaaaaATGAAGACTAATCTTTTTCTCTTTCTCATCTTTTCACTTCTCCTATCATTATCCTCGGCCGAATTCGATGTCCAGTTACAGGAGTCAGGCGGGGGTCTGGTCCAGCCCGGAGGCAGCCTGCGTTTGTCCTGTGCAGCTTCAGATCTTGGGCTATATACATACGCCATAGGGTGGTTCAGGCAAGCTCCAGGGAAAGAATATGGCACCGATGGGAGGGTTTCATATTCAGAGTGCTCAATCTCCAGTGCTGACTCCGTTAAGGGCCGATTCACTACAAGCCGTGACAATGCCAAAAATACAGTCTACCTGCAGATGAATAGCCTCAAACCCGAAGACACTGCTGTGTACTATTGCGCCTACTATTATTATTGCCCAGGTATGTACTATACAGGCACCACCGTAGGTTCCCACGATGCCCCAAGCTGGGGGAAAGGTACACAAGTAACTGTCAGCAGCCATCACCATCATCACCACTAGTGATAActagtgatcgttcaaacatttggcaataaagtttcttaag

>SP-CoV19_his-GFP

tggtttttatttttaattttctttcaaatacttccatcccgggaaaaaATGAAGACTAATCTTTTTCTCTTTCTCATCTTTTCACTTCTCCTATCATTATCCTCGGCCGAATTCGATGTCCAGTTACAGGAGTCAGGCGGGGGTCTGGTCCAGCCCGGAGGCAGCCTGCGTTTGTCCTGTGCAGCTTCAGGGCTAACACTTGATTACTATGCCATAGGGTGGTTCAGGCAAGCTCCAGGGAAAGAAAGGGAGGGGGTTAGTTGCATCTCATCCTCAGATGGCTCAACCTATTATGCTGACTCCGTTAAGGGCCGATTCACTACAAGCCGTGACAATGCCAAAAATACAGTCTACCTGCAGATGAATAGCCTCAAACCCGAAGACACTGCTGTGTACTATTGCGCCGCCGTACCATCCACCTATTACAGCGGCACATACTATTATACCTGCCACCCAGGTGGTATGGATTATTGGGGGAAAGGTACACAAGTAACTGTCAGCAGCCATCACCATCATCACCACGGATCCGGAGCAACCAACTTTTCTCTCTTGAAACAAGCCGGTGATGTTGAGGAAAATCCTGGCCCCGTGAGCAAGGGCGAGGAGCTGTTCACCGGGGTGGTGCCCATCCTGGTCGAGCTGGACGGCGACGTAAACGGCCACAAGTTCAGCGTGTCCGGCGAGGGCGAGGGCGATGCCACCTACGGCAAGCTGACCCTGAAGTTCATCTGCACCACCGGCAAGCTGCCCGTGCCCTGGCCCACCCTCGTGACCACCCTGACCTACGGCGTGCAGTGCTTCAGCCGCTACCCCGACCACATGAAGCAGCACGACTTCTTCAAGTCCGCCATGCCCGAAGGCTACGTCCAGGAGCGCACCATCTTCTTCAAGGACGACGGCAACTACAAGACCCGCGCCGAGGTGAAGTTCGAGGGCGACACCCTGGTGAACCGCATCGAGCTGAAGGGCATCGACTTCAAGGAGGACGGCAACATCCTGGGGCACAAGCTGGAGTACAACTACAACAGCCACAACGTCTATATCATGGCCGACAAGCAGAAGAACGGCATCAAGGTGAACTTCAAGATCCGCCACAACATCGAGGACGGCAGCGTGCAGCTCGCCGACCACTACCAGCAGAACACCCCCATCGGCGACGGCCCCGTGCTGCTGCCCGACAACCACTACCTGAGCACCCAGTCCAAGCTGAGCAAAGACCCCAACGAGAAGCGCGATCACATGGTCCTGCTGGAGTTCGTGACCGCCGCCGGGATCACTCTCGGCATGGACGAGCTGTACAAGTAGTGATAActagtgatcgttcaaacatttggcaataaagtttcttaag

>SP-his_CoV19-GFP

tggtttttatttttaattttctttcaaatacttccatcccgggaaaaaATGAAGACTAATCTTTTTCTCTTTCTCATCTTTTCACTTCTCCTATCATTATCCTCGGCCGAATTCCATCACCATCATCACCACGATGTCCAGTTACAGGAGTCAGGCGGGGGTCTGGTCCAGCCCGGAGGCAGCCTGCGTTTGTCCTGTGCAGCTTCAGGGCTAACACTTGATTACTATGCCATAGGGTGGTTCAGGCAAGCTCCAGGGAAAGAAAGGGAGGGGGTTAGTTGCATCTCATCCTCAGATGGCTCAACCTATTATGCTGACTCCGTTAAGGGCCGATTCACTACAAGCCGTGACAATGCCAAAAATACAGTCTACCTGCAGATGAATAGCCTCAAACCCGAAGACACTGCTGTGTACTATTGCGCCGCCGTACCATCCACCTATTACAGCGGCACATACTATTATACCTGCCACCCAGGTGGTATGGATTATTGGGGGAAAGGTACACAAGTAACTGTCAGCAGCGGATCCGGAGCAACCAACTTTTCTCTCTTGAAACAAGCCGGTGATGTTGAGGAAAATCCTGGCCCCGTGAGCAAGGGCGAGGAGCTGTTCACCGGGGTGGTGCCCATCCTGGTCGAGCTGGACGGCGACGTAAACGGCCACAAGTTCAGCGTGTCCGGCGAGGGCGAGGGCGATGCCACCTACGGCAAGCTGACCCTGAAGTTCATCTGCACCACCGGCAAGCTGCCCGTGCCCTGGCCCACCCTCGTGACCACCCTGACCTACGGCGTGCAGTGCTTCAGCCGCTACCCCGACCACATGAAGCAGCACGACTTCTTCAAGTCCGCCATGCCCGAAGGCTACGTCCAGGAGCGCACCATCTTCTTCAAGGACGACGGCAACTACAAGACCCGCGCCGAGGTGAAGTTCGAGGGCGACACCCTGGTGAACCGCATCGAGCTGAAGGGCATCGACTTCAAGGAGGACGGCAACATCCTGGGGCACAAGCTGGAGTACAACTACAACAGCCACAACGTCTATATCATGGCCGACAAGCAGAAGAACGGCATCAAGGTGAACTTCAAGATCCGCCACAACATCGAGGACGGCAGCGTGCAGCTCGCCGACCACTACCAGCAGAACACCCCCATCGGCGACGGCCCCGTGCTGCTGCCCGACAACCACTACCTGAGCACCCAGTCCAAGCTGAGCAAAGACCCCAACGAGAAGCGCGATCACATGGTCCTGCTGGAGTTCGTGACCGCCGCCGGGATCACTCTCGGCATGGACGAGCTGTACAAGTAGTGATAActagtgatcgttcaaacatttggcaataaagtttcttaag
